# RefPrimeCouch—a reference gene primer CouchApp

**DOI:** 10.1093/database/bat081

**Published:** 2013-12-24

**Authors:** Jascha Silbermann, Catrin Wernicke, Heike Pospisil, Marcus Frohme

**Affiliations:** ^1^Department of Molecular Biotechnology and Functional Genomics, University of Applied Sciences Wildau, 15745 Wildau, Germany and ^2^High Performance Computing in Life Sciences/Bioinformatics, University of Applied Sciences Wildau, 15745 Wildau, Germany

## Abstract

To support a quantitative real-time polymerase chain reaction standardization project, a new reference gene database application was required. The new database application was built with the explicit goal of simplifying not only the development process but also making the user interface more responsive and intuitive. To this end, CouchDB was used as the backend with a lightweight dynamic user interface implemented client-side as a one-page web application. Data entry and curation processes were streamlined using an OpenRefine-based workflow. The new RefPrimeCouch database application provides its data online under an Open Database License.

**Database URL:** http://hpclife.th-wildau.de:5984/rpc/_design/rpc/view.html

## Introduction

The new RefPrimeCouch database application was required for a quantitative real-time polymerase chain reaction (qPCR) standardization project. The goal was to build a system for the efficient storage and retrieval of human reference genes for different tissues. Reference genes are important for the standardized selection of primers for high-quality qPCR procedures.

The new database was to be similar in functionality to existing reference gene primer databases, such as RT-PrimerDB (1). However, the ability to carry out a tissue-specific reference gene search was missing from existing solutions and was to be included in the newly developed system.

### Complexity of data domain

Before beginning the actual work of designing and building a new database application, the complexity of the data domain should be assessed. This is done to ensure that the methodology chosen conforms to the problem at hand in the most optimal and efficient manner. For example, not every database application requires scalability across multiple orders of magnitude.

The expected magnitude of the data store can be estimated well, as the total number of human genes is known to be limited. The maximum number of gene entries can thus be estimated to be in the 10^4^ range, quite small by modern standards. This number represents an upper limit to the total number of entries possible and, in practice, only the order of 10^2^–10^3^ genes are likely required to be held in the database.

For each gene, the data to be stored include gene-specific information and associated primer data, with multiple primers per gene. As part of the standardization effort, each set of primer data also had to be backed up by a publication reference. From a preliminary data set, it could be concluded that for a gene with a single set of primer information, some ≥30 fields of data had to be stored.

### RefPrimeCouch development methodology

Development of the new database application was carried out as a joint project between molecular biology researchers and a bioinformaticist. The researchers collected sample data in a spreadsheet, while the bioinformaticist designed and built the new database application.

**Figure 1.**
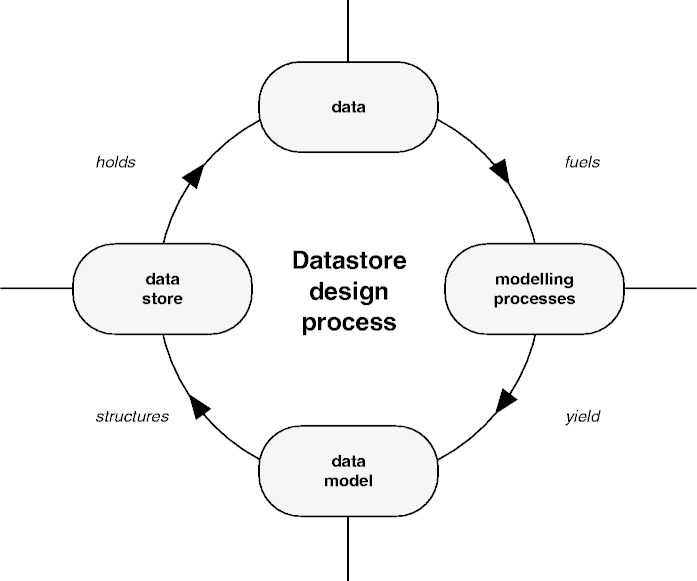
The data store design process is shown. Modeling processes yield a data model, which is subsequently used to structure a data store. In the traditional (relational) approach, definition of a rigid data model (schema) is required to structure the data store. The more agile schema-less approach allows for multiple iterations through the design process, thus making it possible to incrementally improve the data store structure.

Figure 1 shows the general data store design process. This usually presents somewhat of a ‘chicken and egg’ problem, as a sample set of data is needed to begin the process of designing an abstract data model. The starting data have to be stored somewhere—assuming they are of any substantial size—but no specialized data store exists at this point. Traditionally, one would be required to design a rigid database schema as a first step toward developing the new data store.

As neither the final requirements for the database application nor the final data model were known at the beginning of the project, a more ‘agile’ development methodology—compare (2)—was chosen to allow for incremental improvement of the new system through multiple iterations. As a key component of this agile methodology, a schema-less database was used.

Schema-less database solutions, often grouped together under the umbrella term ‘NoSQL’ to differentiate them from the mainstream relational database management systems (RDBMS), have come into widespread use in recent years. They allow for great development flexibility, as the data model can be continually adjusted with relative ease throughout the entire development process.

A design theme of ‘standardization of methods and data, simplicity of resulting systems and processes and improvement over existing solutions’ was chosen to provide an overarching structure for the work to be carried out while still allowing for the required flexibility and fluidity of decision-making along the way.

## Methods

### standardization

#### Data model, data format and database license

The Real-time PCR Data Markup Language (RDML) consortium (3) publishes the minimum information for publication of quantitative real-time PCR experiments (MIQE) guidelines (4) to provide a consensus format for qPCR experiments data. Because our intent was not to record experimental data, but to supply a standardized data set as the basis for new experiments, only a subset of the data items noted in the guidelines was used as the basis for our data model. Most importantly, we borrowed the term ‘target’ to denote a tissue-specific set of qPCR information. Tissue-specific targets are stored for each gene, encompassing information on primers, qPCR preparation, qPCR conditions and a publication reference.

The JavaScript Object Notation (JSON) format (5) was chosen as the universal web-enabled data format used on all levels throughout the project. Using JSON, it is possible to describe hierarchical objects of almost arbitrary complexity in a simple manner. JSON is one of the most widely used formats and can be used to model data structures, store actual data and exchange data between programs and platforms.

The Open Database License (ODbL) (6) was chosen for publication of all reference gene data.

#### Web application components

To allow users to interface with the database and the data contained therein, a web application was developed. To build the web application, a number of standardized components were used. The popular HTML5 Reset (7) template was used as the basis for the web application HTML5 + CSS code.

The initial data model and the database views were written in the JavaScript (8) programming language. The jQuery (9) library was used to provide cross-browser scripting capabilities, offering dynamic data updates on user interaction and allowing the use of plug-ins to interface with third-party systems. The JSON format was ubiquitously used for data storage and exchange.

The new web application was built as a single-page CouchApp, the overall structure of which was inferred from (10). This can serve as a solid starting point for similar projects as well.

#### Apache CouchDB

Apache CouchDB (11) is a modern document-oriented database. Documents in this context are self-contained hierarchical sets of data, represented in the JSON format. Documents are automatically versioned, and each document can be accessed via a unique address (URL). Furthermore, besides data in the JSON format, files of any type may be stored as ‘attachments’.

Each document can be accessed in its entirety via its URL. To access sets of values from multiple documents over a specific field, ‘views’ are created and stored along with the database. Views follow the map/reduce pattern (12), filtering data by a set of specified criteria. Views are implemented as simple JavaScript functions and can be accessed from within the web application via the CouchDB jQuery plug-in.

Apache CouchDB ships with a built-in management and administration interface called Futon. This provides an easy way to manage databases and users and can also be employed to quickly add and modify individual documents. An interesting feature of Futon is the replicator, which can be used to duplicate and sync databases between different CouchDB installations.

#### OpenRefine

OpenRefine (13), a ‘free open source power tool for working with messy data’ can import tabular data in all major formats, including XLS, ODS and CSV. OpenRefine supplies a host of advanced features not normally found in spreadsheet-based applications. Most operations in OpenRefine can be carried out in bulk on entire columns or rows of data, and operations may be extracted as code for refinement and reuse.

The ‘facet’ features allow grouping of row or column values and can be used to gain a high-level overview of data variance and to find outliers and homogenize data. Batch editing is code-based, allowing for easily replicable semi-automated workflows. Apart from an internal expression language called ‘GREL’, Jython and Closure may be used for coding.

OpenRefine also includes powerful automated export mechanisms. Using the templating feature, it is possible to convert tabular data into hierarchical formats, such as JSON, HTML or Wiki markup code, with a minimum of effort.

## Results

### simplicity of systems and processes

#### Data content and data model

The database currently comprises information on 81 distinct primers for 40 reference genes expressed in 57 tissues. Approximately 30 fields of data are stored for every gene, including the gene symbol, full name, list of synonyms and RefSeq ID. Targets for each gene include gene- and tissue-specific qPCR information backed by a publication reference, linked to via PubMed.

A core of 48 frequently cited recent publications was used to assemble the primary data set. Publications were from the years 2004 through 2012, with the majority of 30 publications being from the years 2008 through 2012 and the remaining 18 publications being from the years 2004 through 2007.

The gene viewer can be accessed via the following URL: http://hpclife.th-wildau.de:5984/rpc/_design/rpc/view.html

The official gene symbol as provided by the HUGO Gene Nomenclature Committee (HGNC) (14) is used as the unique identifier for reference gene documents. Therefore, it is possible to both reference and retrieve the JSON data for any reference gene in RefPrimeCouch by appending the HGNC gene symbol to the CouchApp database URL:

http://hpclife.th-wildau.de:5984/rpc/

For example, the link ‘http://hpclife.th-wildau.de:5984/rpc/ATP5B’ will point to the latest revision of the document referring to the gene with the official symbol ‘ATP5B’ (‘ATP synthase, H+ transporting, mitochondrial F1 complex, beta polypeptide, mRNA’). The latest revision number is included in the document.

**Figure 2.**
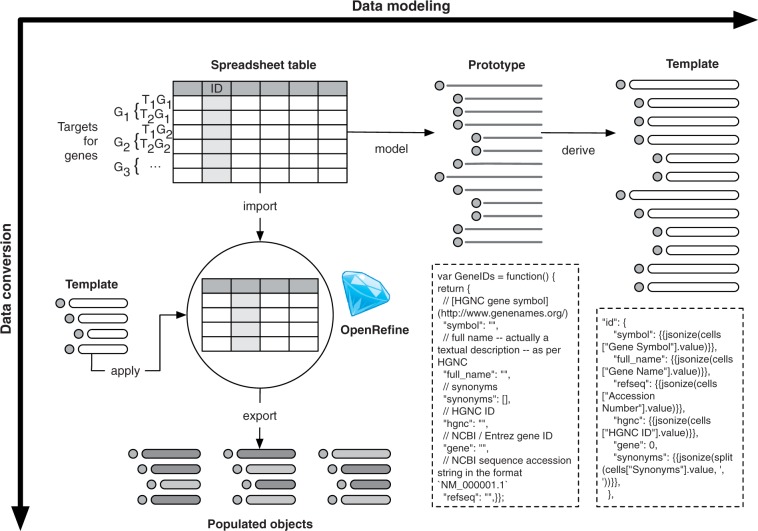
The processes of data modeling and data conversion are shown along the horizontal and vertical axes, respectively. To create the data model, a spreadsheet with sample data was used as a basis to model a prototype data structure. From this prototype, template code for the automated conversion of the source data was derived; sample code for the prototype definition and the data conversion template is shown. The data conversion workflow incorporates the template code, which is fed into OpenRefine along with the data in a tabular format. Using OpenRefine’s templating mechanism, the tabular data are exported as a set of populated objects in the JSON format. The objects share a common hierarchical structure, but comprise different values, as denoted by their shading.

Figure 2 shows the data modeling and subsequent data conversion steps involved in transferring the data collected by the researchers into the new data store.

First, a prototype data model was specified using JavaScript. From this prototype, templates in various formats were derived. Each template serves a specific role in the application development and data conversion workflows. For example, an HTML template was built to visualize the data, whereas an OpenRefine export template was constructed for the automated transformation of tabular data held in standard spreadsheet into JSON objects.

#### Web application and user interface

RefPrimeCouch was built as a single-page web application, meaning that selected data are dynamically presented on a single page without the need to reload the page to change data. For increased efficiency, advantage was taken of the constrained size of the data domain: only a single request is sent to the database backend to fetch the entire set of available genes. This data set is then cached in the user’s browser for the duration of the interaction, which makes the user interface responsive and reduces server load to a bare minimum.

The visual user interface consists of a gene viewer taking up the majority of screen real estate, as well as a gene list and a set of filters, and controls on the right side. The gene viewer provides a hierarchical view of a selected gene with all the associated information; the gene’s identity is prominently shown at the top of the page, followed by multiple targets comprising fine-grained qPCR details in a tabular representation. The gene list shows all the genes currently available in the database.

The filters allow the list of all available genes to be narrowed down according to various search criteria. It is possible to textually constrain genes by gene identity, including the gene’s official symbol, synonyms and full name. Furthermore, pseudogenes may be excluded from the list, and genes can be constrained to those containing targets for a specific tissue only. Thus, it is possible to quickly find a set of genes with targets for tissues such as ‘cancer’, ‘pancreas’, ‘carcinoma’, ‘adipose’, ‘blood’, ‘muscle’, and ‘brain’.

To facilitate interaction with other major online resources, external sites with further information are linked to. These include the HGNC (14) for the gene symbol information, the NCBI for the RefSeq entry (15) and the taxonomy browser and GeneCards (16) for the chromosomal location image. Furthermore, the entire document in the JSON format is available under a link as well.

#### Data collection and curation workflow

Data for all genes and their associated targets were collected by multiple researchers in a shared spreadsheet file. The column structure of the spreadsheet was refined multiple times over the course of the project to more accurately reflect the data domain. All data were pulled from available publications and are hence backed up by a publication reference.

**Figure 3.**
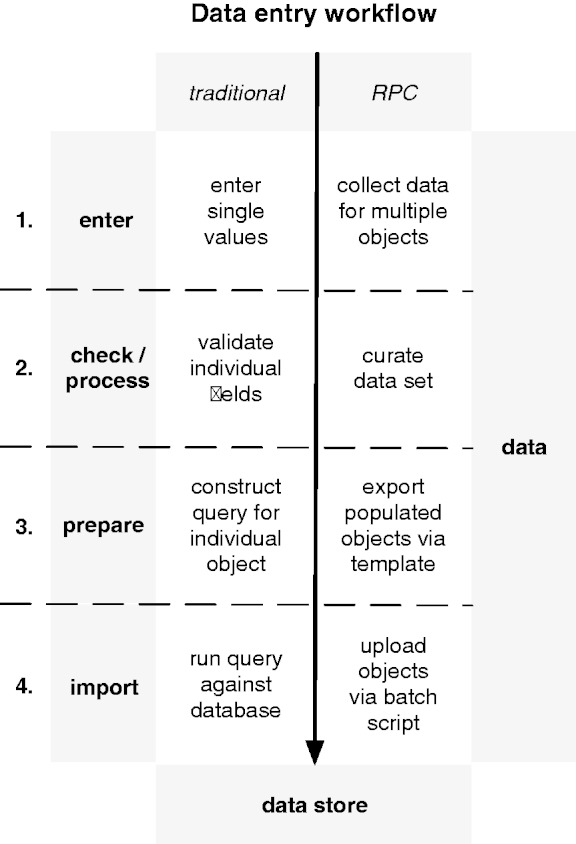
The traditional data entry workflow is compared with the RefPrimeCouch (RPC) workflow. Data entry comprises four steps, three of which (steps 2 and 4) are machine-driven in the traditional workflow and take place without human interaction. The novel workflow involves a code-supported human curator at every single step.

The entire set of gene–target data was first manually curated by the researchers within the spreadsheet. When the data were complete from the researchers’ point of view, the entire spreadsheet was loaded into OpenRefine for further automated processing. Here, further curation and normalization steps, such as checking for redundant and missing values, were carried out. On completion of the data processing steps, the tabular data were exported into a hierarchical structure in the JSON format and uploaded to the database via a script. See Figure 3 for a detailed explanation of the steps comprising the data entry workflow.

## Discussion

### improvement over existing solutions

#### CouchApp improves development process

One of the main advantages of the CouchApp-based approach is the high level of flexibility that can be attained during development and maintenance.

This way it is possible to support the diverse—and often changing—biological requirements with an extensible database application that can grow and adapt with evolving needs, while being much easier to maintain and develop than would be possible using the more rigid traditional development process.

The established methodology for developing a database-driven web application involves a three-tier architecture:

1. Database layer: RDBMS + database scheme (SQL)
2. Application layer: PHP^1^ (or another programming language running on the web server, such as Java, Python and Perl) + SQL statements/ORM^2^ (ORM = object-relational mapping, a technology to store hierarchical objects in a RDBMS)
3. Presentation layer: HTML, CSS + JavaScript

Using a CouchApp-based approach, this could be streamlined to the following two-tiered model:

1. Database layer: CouchDB + views (JavaScript)
2. Application and Presentation layer: HTML, CSS + jQuery

Note that two entire levels of technology are removed, namely, SQL and PHP. These are the most ‘static’, least amenable to agile development practices. Therefore, the CouchApp-based approach helps to greatly simplify the development process.

#### OpenRefine improves curation

Many existing databases use web forms to allow users to submit new data. Normally, this involves the user filling out individual fields for each set of data, followed by an automatic validation step of the entered values before submission (compare Figure 3). This is done to prevent the user from entering non-sensical or incomplete values into the database.

The problem here is that the efficiency of the input procedure is limited by the design of the web form. Badly designed forms and input validation algorithms may force the user to perform many steps redundantly, which can be frustrating. Furthermore, because the validation algorithms must work for any possible input, it can be difficult to check for more than the most basic types of mistakes without too severely restricting users.

Using OpenRefine to curate multiple sets of data before uploading to the database completely solves the problems associated with web form-based data entry. Researchers are free to assemble multiple sets of data in a standardized spreadsheet file, which is then processed by the database curator. Processing in OpenRefine is code-driven, and operations are carried out on multiple values at once, making for highly efficient and reproducible workflows. Furthermore, the code used can be published for validation and reproduction of curation procedures.

Because the curation steps are performed by a human being and multiple sets of data are included at the same time, the curator can draw on shared information between data sets to enforce higher-order quality standards. For example, similar values across data sets can be detected and merged using the ‘facets’ feature. Automatic validation of data, on the other hand, works on the single field level only.

Even though human interaction is required using an Open-Refine-based curation approach, no efficiency is lost, as multiple sets of data are processed at once. Furthermore, because no dedicated web interface is required for data upload and curation, no resources have to be spent on development and maintenance. Instead, a clever curator can continually improve the code used to drive the curation steps within OpenRefine.

#### RefPrimeCouch improves user experience

Using RefPrimeCouch, it is possible to perform a search for primers for stably expressed genes, i.e. genes that are not regulated, in a given tissue. Furthermore, this search can be extended to different tissues, which can be especially useful to cover the course of a disease affecting multiple tissues over time. For some applications, it can also be interesting to see whether a certain gene that is regulated in one tissue can be used as a reference gene in a different tissue.

A database whose content is primarily organized along primers is necessarily strongly restricted in its usefulness for the purpose of finding tissue-specific reference genes as described above. Furthermore, a reference gene primer that is regulated in a certain tissue may appear to not function correctly in a different one and may thus be excluded from such a database altogether.

Additionally, the search functionality of some major existing primer databases is lacking in usability. To name one example, RTPrimerDB (1) uses its own ID scheme to identify database entries, which is of little use to a researcher. Alternatively, the official gene symbol may be used to query the database.

RefPrimeCouch, on the other hand, uses the official gene symbol as the internal ID, removing an unnecessary layer of indirection between the user and the data. Furthermore, the database may be queried not only by the gene’s symbol but also by synonym and full name, allowing for a much more exploratory approach to searching.

Traditionally, users access data from online database resources by filling out a web form and submitting their search. Search criteria specification and refinement then is a two-step process, which can be frustrating when searches turn up empty or unspecific results. Because no direct feedback is given, it is also difficult to quickly compare results for multiple search queries.

Using RefPrimeCouch, modification of search criteria yields instantaneous results, thus making it easy to quickly find the right criteria for a desired set of data. This also simplifies the process of comparing results for different searches, as criteria may be toggled on or off with immediate feedback to the user.

**Figure 4.**
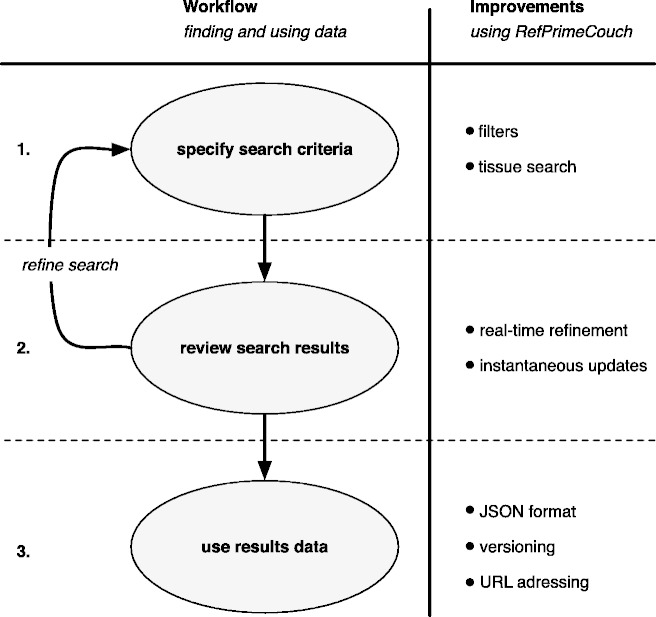
The general workflow for finding and using data is shown, along with the improvements afforded by RefPrimeCouch. Users typically specify, review and refine search criteria iteratively (steps 1 and 2)] to arrive at a desired data set [step 3]. RefPrimeCouch offers improvements over existing solutions at all three steps. Instead of using a form to be filled out for the search, users specify search criteria using filters. As a further improvement, users may restrict their search to genes that are known to be usable as reference genes within a certain tissue [step 1]. Not only can the refinement be carried out in real-time, without any page reloads, the results of the refinement are instantaneously shown to the user. The instantaneous feedback allows users to reach the optimal search criteria quickly; this feature also instantly alerts the user of ‘empty’ search results [step 2]. Once the desired data have been located within the data store with appropriate search criteria, the usefulness of the thus obtained data is potentially limited by the capabilities of the underlying system. The data yielded by a request to RefPrimeCouch are highly usable in a scientific context, as data can easily be cited and reused.

See Figure 4 for a detailed description of the improvements afforded by using RefPrimeCouch.

## Conclusion

We are not the first to develop a CouchDB-based biological database (17). However, what sets our approach apart is the development methodology, which aims to incorporate high-level goals such as simplicity, standardization and improvement throughout all levels of the project.

Our methodology is applicable to any curated database of small to modest size (‘hundreds to thousands’ instead of ‘hundreds of million’ entries) and is especially well suited to exploratory projects with limited development capacities.

Although the current RefPrimeCouch web interface is a promising first step toward greatly enhanced usability, much work could still be invested into this particular aspect of the application. It would be desirable to rebuild the application and presentation layer using a framework, such as (18–20). This should yield enhanced browser support and user experience, such as providing direct links into the gene viewer from URLs.

Currently, the interface has been tested and works with modern, standards-compliant browsers based on the Gecko, Webkit and Blink browser engines.

Another interesting area of ongoing development is the continual improvement of the existing data curation workflow to allow easy integration of additional gene and target data. To this end, a standardized spread sheet will be made available for other scientists to use for contribution of reference gene primer data to the RefPrimeCouch project.
